# The role and therapeutic potential of stem cells in skeletal muscle in sarcopenia

**DOI:** 10.1186/s13287-022-02706-5

**Published:** 2022-01-24

**Authors:** Zijun Cai, Di Liu, Yuntao Yang, Wenqing Xie, Miao He, Dengjie Yu, Yuxiang Wu, Xiuhua Wang, Wenfeng Xiao, Yusheng Li

**Affiliations:** 1 Department of Orthopedics, Xiangya Hospital, Central South University, Changsha, 410008 Hunan China; 2 National Clinical Research Center for Geriatric Disorders, Xiangya Hospital, Central South University, Changsha, 410008 Hunan China; 3 School of Kinesiology, Jianghan University, Wuhan, 430056 China; 4 Xiang Ya Nursing School, Central South University, Changsha, 410008 Hunan China

**Keywords:** Sarcopenia, Muscle-derived stem cells, Satellite cells, Muscle stem cells

## Abstract

Sarcopenia is a common age-related skeletal muscle disorder featuring the loss of muscle mass and function. In regard to tissue repair in the human body, scientists always consider the use of stem cells. In skeletal muscle, satellite cells (SCs) are adult stem cells that maintain tissue homeostasis and repair damaged regions after injury to preserve skeletal muscle integrity. Muscle-derived stem cells (MDSCs) and SCs are the two most commonly studied stem cell populations from skeletal muscle. To date, considerable progress has been achieved in understanding the complex associations between stem cells in muscle and the occurrence and treatment of sarcopenia. In this review, we first give brief introductions to sarcopenia, SCs and MDSCs. Then, we attempt to untangle the differences and connections between these two types of stem cells and further elaborate on the interactions between sarcopenia and stem cells. Finally, our perspectives on the possible application of stem cells for the treatment of sarcopenia in future are presented. Several studies emerging in recent years have shown that changes in the number and function of stem cells can trigger sarcopenia, which in turn leads to adverse influences on stem cells because of the altered internal environment in muscle. A better understanding of the role of stem cells in muscle, especially SCs and MDSCs, in sarcopenia will facilitate the realization of novel therapy approaches based on stem cells to combat sarcopenia.

## A brief introduction to sarcopenia

Sarcopenia, from the Greek roots sarx (flesh) and penia (loss), was coined for the first time in 1989 by Irwin Rosenberg and was initially defined as a disease closely related to aging that featured continuous progression and generalized loss of skeletal muscle mass and function [1]. Sarcopenia is a commonly occurring but obscure problem in the population that is older than middle age, which causes mobility limitations and increases the risk of fracture, fall, and mortality [2]. With disease progression, diverse clinical manifestations present, and sarcopenia may be considered a geriatric syndrome rather than an isolated disease because of its systematic effects. At present, the most popular definition and diagnostic criteria were proposed by the European Working Group on Sarcopenia in Older People (EWGSOP2) in 2019 [3], with the aim of increasing awareness of sarcopenia and its risk among clinical practitioners. In clinical work, lumbar or thoracic computed tomography (CT) images are useful for body composition assessment and reveal high rates of sarcopenia [4].

## The etiology of sarcopenia

The etiology of sarcopenia is multifactorial, involving multiple interacting factors, such as insufficient activity that is closely associated with aging, elevated inflammatory factors, neuromuscular junction degeneration, hormone imbalance, malnutrition, and gene regulation [2, 5–7]. Eventually, some or all of these changes can result in sarcopenia through myocyte apoptosis [2]. Of these factors, aging is of the greatest importance in the onset and progression of sarcopenia, which can lead to an imbalance between anabolism and catabolism of muscle tissue and then lead to the loss of muscle, with a notable decrease in the size and number of type II myofibers [2]. Mitochondrial dysfunction plays a key role in muscle atrophy, as well as changes in multiple pathways [8]. It is worth noting that sarcopenia may be closely related to the skeletal system because there is intimate cross-talk between muscles and bones that is mediated by multiple endocrine factors [9]. Elevated 15-hydroxyprostaglandin dehydrogenase (15-PGDH), the prostaglandin E2 (PGE2)-degrading enzyme, could serve as a hallmark of aged skeletal muscle tissue [10]. Palla et al. showed that inhibition of 15-PGDH increased aged muscle mass, strength, and exercise performance, suggesting that 15-PGDH may become a potential therapeutic target in future to counter the debilitating muscle atrophy characteristic of sarcopenia [10]. Lv et al. [11] characterized a novel functional lncRNA designated lncMGPF (lncRNA muscle growth promoting factor) and reported their new findings that lncMGPF is a positive regulator of muscle growth and regeneration in mice, as lncMGPF knockout significantly reduced muscle mass and impaired muscle regeneration, which indicates the possibility of treating sarcopenia at the genetic level. Although some mechanisms of sarcopenia occurrence have been elucidated, the pathogenesis remains complex and incompletely understood, which allows room for further studies in future. Although, the current understanding of sarcopenia is much deeper than before, there are currently no widely recognized treatment guidelines for sarcopenia. Sarcopenia is difficult to treat once it occurs due to its complexity and property as a risk factor for other diseases. Therefore, for elderly individuals who are prone to sarcopenia, it is crucial to develop a good primary and secondary prevention system to avoid risk factors and achieve early identification and subsequent intervention.

## Stem cells in muscle

Skeletal muscle is a kind of voluntary and striated muscle. Skeletal muscle is attached to the skeleton and contracts or relaxes under the control of somatic motor nerves, allowing the whole body to move at will. Because it has the greatest mass, skeletal muscle tissue, which is mainly comprised of water, plays an important role in the human body and accounts for approximately 40% of the body weight [12]. Regrettably, skeletal muscle defects or injuries caused by congenital factors, tumors, primary myopathy, metabolic diseases and other reasons are common in the clinic. Therefore, as an important reparative cell in muscle when lesions occur, stem cells have drawn great attention from researchers and may always be a research hotspot. Because there is still controversy in the medical community regarding the description and classification of all stem cells that are already found in skeletal muscle, we temporarily assumed that there are at least two kinds of stem cells present in skeletal muscle tissue: SCs and MDSCs.

### SCs

In 1961, Mauro first discovered SCs in the muscle of frogs and many other vertebrates by electron microscopy [13]. These SCs, unlike ordinary muscle cells, are able to regenerate repeatedly throughout life. Moreover, these cells do not act as functional units but provide some necessary components for the repair and reconstruction of damaged parts. Since then, SCs have been gradually considered a source of skeletal muscle repair ability. SCs are typically quiescent mononucleated myogenic cells located between the sarcolemma and basement membrane of terminally differentiated myofibers. In addition to ensuring homeostasis, SCs are important initiators and participants in tissue repair when damage occurs [14]. Once exposed to trophic or injury signals from the extracellular matrix (ECM) or after interacting with inflammatory cells, SCs enter the cell cycle and begin to proliferate and differentiate, which are the processes of SC activation. A portion of activated SCs will differentiate into myoblasts to replenish myofibers and eventually repair muscle tissue, while others will self-renew and return to the niche to maintain a normal number of stem cells.

SCs express a series of identifying markers located on the surface membrane, such as caveolin-1 (CAV1), integrin alpha7 (ITGA7) and calcitonin receptor (CTR), regardless of whether they are quiescent or activated [15]. For markers in the nucleus, the transcription factors paired-box 3 (PAX3) and paired-box 7 (PAX7) both have important roles in myogenic specification [14]. PAX7 is continuously and highly expressed and is therefore the best marker, and MRFs have received more attention for their key function during the lifetime of satellite cells, while PAX3 is usually expressed at low levels. MRFs include MYF5, MYOD, MRF4 and MYOG (myogenin), the first three of which are not expressed in myotubes formed from fused myoblasts, but all MRFs are expressed in myoblasts that differentiate from activated satellite cells [16]. Myogenin (Myog) plays a crucial role in adult muscle growth and stem cell homeostasis, which is manifested in controlling myocyte fusion and impacting niche relationships [17].

### MDSCs

As early as 1999, Jackson et al. [18] found high numbers of stem cells with hematopoietic differentiation abilities that expressed SCA-1 and c-Kit in the skeletal muscle of adult mice that were different from SCs due to the absence of commitment to myogenic differentiation. From current evidence, we can conclude that these cells are called MDSCs. It is believed that the presence of MDSCs with multidirectional differentiation ability is based on the fact that skeletal muscles always show regenerative capacity when damaged by recruiting cells that belong to different lineages to complete the regeneration process. Many previous studies have demonstrated that MDSCs, which are pluripotent stem cells, can not only differentiate into mesodermal cell types such as myoblasts, chondrocytes, cardiomyocytes, and hematopoietic lineages but also break the restriction to the germ layer and differentiate into ectodermal and endodermal cell types under appropriate conditions [19, 20]. A recent study showed that SCA-1, CD34, and CD45 may be reasonable markers for the identification of MDSCs [21]. It is generally established that MDSCs express the mesenchymal stem cell markers CD73, CD90, and CD105 [22], and some other positive markers have been revealed, including CD29, CD44, and CD133 [23–26].

**Fig. 1:**
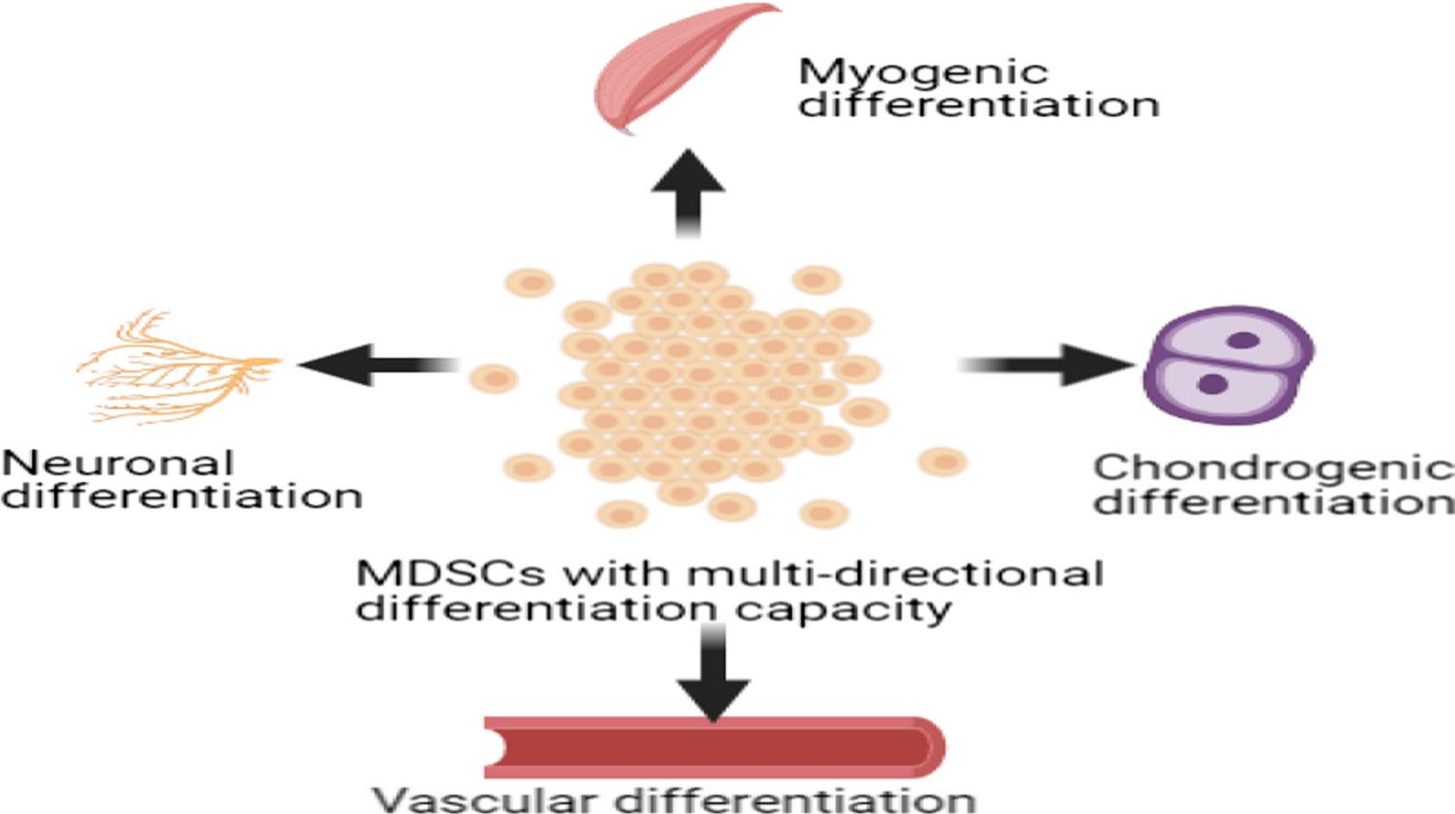
MDSCs display a greater multi-directional differentiation capacity in contrast with SCs

In recent years, MDSCs have shown great promise for the treatment of multiple diseases. First, similar to satellite cells, MDSCs can be used to repair muscle damage, indicating the therapeutic potential for sarcopenia. They can be expanded in vitro for up to 30 passages while maintaining myogenic potential. Mitutsova et al. [27] revealed that MDSCs could combat diabetes arising from pancreatic beta cell deficiency by investigating the effects of MDSCs on diabetic mouse models. The high survival rate and superior chondrogenic differentiation capacity of these cells make MDSCs plausible candidates for articular cartilage repair [20]. The expression levels of the smooth muscle cell-specific contractile proteins smooth muscle α-actin and calponin were dramatically increased after treatment with TGF-β1 in the MDSC population, suggesting hope for the treatment of diseases resulting from smooth muscle defects such as stress-induced urinary incontinence and pelvic organ prolapse [21, 28]. The ability of MDSCs to promote functional healing of ligaments offers the possibility for the treatment of disorders in sports medicine [29]. Examples of MDSC differentiation are shown in Fig. 1. There are various studies on MDSCs currently underway. In summary, existing research findings together with the strong self-renewal ability and high survival rate of autografts after transplantation show that MDSCs have broad application prospects in TE and stem cell-mediated therapy.

### Insights into the differences and associations between MDSCs and SCs

Although, the scientific community has always thought that muscle stem cells refer to SCs, our perspective is that, rigorously speaking, the term “muscle stem cells” should include not only SCs but also all the other stem cell populations present in muscle, such as MDSCs. Completely equating muscle stem cells with SCs is slightly inappropriate. In other words, it is just a habitual reference. From the introduction to SCs and MDSCs described above, we can conclude that there are differences as well as connections between these two cell types.

There are a number of marked distinctions between MDSCs and SCs. PAX7 serves as an indicator of SC functions and is the best identifying marker of SCs, but PAX7 is not adequately expressed in MDSCs [30]. Significant weight loss occurred in PAX7 mutant mice in comparison with wild-type mice, but the proportion of MDSCs was unaffected by PAX7 deletion [31]. The most distinct difference in function between MDSCs and SCs is that the former exhibit multidirectional differentiation abilities, but the latter generally only exhibit myogenic differentiation.

Their common characteristic is their ability to participate in skeletal muscle repair and regeneration. Similar to clusters of SCs with heterogeneous properties, MDSCs, as a general designation for a group of stem cells isolated from muscle with multidirectional differentiation potential, also exhibit heterogeneity [20]. Deasy et al. [32] reported that MDSCs were distinct and could be precursors of SCs in skeletal muscle.

A PubMed search as of November 2, 2021 for “satellite cells” showed 5869 results, while a similar search for “muscle-derived stem cells” yielded 363 results, which indicates the infancy of MDSC research compared to SCs. To better study MDSCs, isolation and purification methods have been reported, and the most common method is the preplate technique. This technique isolates different populations of cells from the skeletal muscle of mice based on their adhesion to type I collagen-coated flasks. However, due to the overlapping marker profiles of MDSCs and SCs, it may be difficult to completely distinguish MDSCs from SCs. In regard to specific discrimination methods of these two cell populations, it is regrettable that the literature has not yet reached a consensus on this point. The characteristics of the two types of stem cells in human muscle are summarized in Table 1.

**Table 1:** Characteristics of SCs and MDSCs

Stem cell source	Major access	Markers in human	Common and respective strengths	
SCs	Muscle biopsy	PAX7, MYF5, MYOD, MYOG, MRF4, CTR, CAV1, ITGA7	Robust proliferation and differentiation potential No toxicity and immune rejection	More investigated
MDSCs	Preplate technique	SCA-1, CD34, CD45, CD73, CD90, CD105, CD29, CD44, CD133		Multilineage differentiation capacity Larger mass accessible

## SCs in sarcopenia

Currently, sarcopenia pathogenesis is not completely clear, but it is clear that stem cells in muscle play an important role in this process. Sarcopenia may be closely related to SC dysfunction. The effect of SCs in repairing muscle tissue through proliferation and differentiation and maintaining muscle mass seems to play an antagonistic role in the onset and progression of sarcopenia. Sarcopenia may be associated with reduced numbers of SCs and the suppression of SC functions, and the alterations in the ECM of muscle tissue that occur in sarcopenia may in turn affect the number and functional state of SCs. These factors may influence each other under disease conditions. While there is a study indicating that the loss of satellite cell-dependent regenerative capacity neither accelerates nor exacerbates sarcopenia [33], we cannot conclude that SCs are not related to sarcopenia at all. Instead, a majority of reports showed an association between them [34, 35].

### The number of SCs

Although, the mainstream view is that during aging, the number of SCs is reduced [36, 37], a minority argue that no significant differences are observed in the number of SCs in young mice and adult mice [38]. Therefore, this review maintains that the changing trend in SC numbers during this process is still controversial. Even so, it is clear that the loss of muscle mass, which is the main characteristic of sarcopenia, is accompanied by a decline in the number of muscle fibers, especially type II fibers [2].

### Differentiation lineages

The maintenance of muscle mass and regeneration abilities critically depends on SC functions, which are increasingly apt to become dysfunctional with aging. Senescence and apoptosis are more likely to occur in aged SCs than in normal SCs [39]. Taylor-Jones et al. [40] cultured and studied myoblasts derived from SCs isolated from mouse hindlimb skeletal muscle and later found that myoblasts from 23-month-old adult mice were more likely to have increased adipogenic potential than those from 8-month-old adult mice regardless of culture conditions. When proliferating, SCs from aged mice can convert from a myogenic to a fibrogenic phenotype [41].

### Mechanisms

One of the most pronounced manifestations of aging is the diminished regenerative capacity of tissues, including muscle tissue, but the specific mechanism of this decline is not fully understood. It is necessary to fully understand the signaling pathways involved in satellite cell senescence, which may provide insights into therapeutic targets for combating sarcopenia. Muscle aging is associated with the revision of homeostasis and a progressive decline in SC functions that is attributed to both extrinsic and intrinsic alterations through many signaling pathways [42, 43]. Here, we list some known mechanisms associated with SC senescence. First, oxidative stress (OS) is an important contributor to defective SC functions and sarcopenia, and there is abundant literature in this regard. OS refers to a state of imbalance between oxidative and antioxidant effects, resulting in neutrophil inflammatory infiltration, increased secretion of proteases, and generation of a large number of oxidative intermediates. OS is a negative effect produced by free radicals and is considered an important reason for aging and disease. Reactive oxygen and nitrogen species (RONS) usually appear after various processes in the human body, and their negative effects are neutralized by antioxidants in normal cases [44]. OS occurs from the imbalance between RONS production and these antioxidant defenses. OS is related to some age-related conditions, including sarcopenia and frailty [44]. Wang et al. [37], by performing bone marrow transplantations between age-mismatched donor and recipient mice, found that aging of the immune system leads to reductions in muscle stem cell populations, promotes their shift to a fibrogenic phenotype, and modulates sarcopenia. Semba et al. [45] revealed that low serum/plasma carotenoids are relevant to low skeletal muscle strength and walking disability. Conboy et al. [46] analyzed injured aged muscle and revealed that SCs were less prone to proliferate and produce the myoblasts necessary for muscle regeneration because of inadequate upregulation of the Notch ligand Delta, which led to impaired muscle regeneration. However, induced upregulation of the Notch ligand could rescue this effect. Thus, we may consider Notch signaling a key determinant of the decline in muscle regenerative ability with age. In addition, there are still other pathways that have been shown to be activated in SCs according to in vivo studies, including JAK/STAT [47], p38 MAPK [48], Wnt [41], and fibroblast growth factor receptor-1 [49], leading to impaired self-renewal capacity. Bosquet et al. [50] described their research involving the p38 MAPK phosphorylation pathway, which is upstream of nuclear factor-kappaB (NF-κB) nuclear translocation and activation, the reduction of which is related to lipid-induced inflammation, and indicated that fatty acid binding protein 4 (FABP4) inhibitors could decrease saturated fatty acid-induced endoplasmic reticulum inflammation in skeletal muscle in diabetes mellitus patients. Therefore, inhibitors may help treat older patients with sarcopenia and diabetes. What is exciting is that ZEB1, which is mediated by p38 MAPK, is indispensable for muscle protection and regeneration [51]. Although, mammalian (or mechanistic) target of rapamycin complex 1 (mTORC1) activity is required for muscle hypertrophy, overactivity of mTORC1 is vital to a majority of processes involved in aging and accelerates sarcopenia [52]. Liu et al. [53] showed that during chronological aging, SCs exhibited reduced transcription of histones due to the acquisition of H3K27me3. Zykovich et al. [54] observed hypermethylation throughout the genome within the aged group compared with the young group. There is no doubt that the loss of SC number and regenerative capacity may result in skeletal muscle dysfunction, volume reduction and eventually sarcopenia. It is worth noting that, in turn, aging can also affect the function and fate of SCs [55]. Accordingly, once sarcopenia is diagnosed early or a propensity for sarcopenia is identified, it should be addressed immediately to prevent this vicious cycle.

### The role of autophagy

All tissues in the body are in dynamic equilibrium, and skeletal muscle tissue is no exception. The maintenance of this balance requires the normal functioning of the autophagic system [56]. Autophagy can be regulated by a wide array of extracellular and intracellular signals, can facilitate the removal of metabolic waste or damaged cellular components and is an important factor in maintaining SC and myofiber function [56, 57]. A reduction in muscle mass, which is the major characteristic of sarcopenia, can be offset to some extent through autophagy [58]. SC senescence is partly due to the inhibition of autophagy in the regenerative phase [59]. In addition, autophagy impairment in skeletal muscle could cause neuromuscular junction degeneration and precocious aging [60]. In sarcopenia, autophagy might be caused by inflammatory signals, resulting in a skeletal muscle phenotype [61, 62]. Autophagy plays a crucial regulatory role in satellite cell quiescence, activation, differentiation and apoptosis, and SCs isolated from aged muscle tend to show fewer autophagy markers [59, 63]. SCs acquire temporary energy replenishment through autophagy, especially when transitioning from a quiescent state to a proliferative state [64]. He et al. [65] demonstrated that exercise could induce autophagy under the regulation of BCL2 in mice. We can conclude that exercise is a reasonable way to activate autophagy to address sarcopenia [66]. We believe that in future, more information concerning the association between SCs and sarcopenia in terms of autophagy will be elucidated.

## MDSCs and sarcopenia

### MDSCs and sarcopenia in relation to lipid accumulation

Fat tissue is involved in longevity and age-related metabolic dysfunction [67]. Aging is often accompanied by changes in the distribution of adipose tissue in the human body, typically from the subcutaneous region to other parts, such as muscle or visceral organs [67]. As one of the histological changes observed in skeletal muscle in sarcopenia, an age-related increase in fat infiltration is often the reason for declines in muscle function [68–70]. The occurrence and development of intramuscular fat infiltration could result in muscle dysfunction. Robles et al. [71] used magnetic resonance imaging (MRI) to observe intramuscular fat infiltration in lower-limb muscles in old adults with chronic obstructive pulmonary disease (COPD) and showed that, in addition to atrophy, fat infiltration is also responsible for impaired muscle function. Takano et al. [72] dissected a large number of elderly cadavers and found that fat infiltration was likely to cause dysfunctions in hip extension and internal rotation. Skeletal muscle and bone share common embryological origins from mesodermal cell populations, and the decline in strength that occurs with aging results from an accumulation of adipose tissue [73]. Accordingly, if MDSCs undergo adipogenic differentiation, fat infiltration in skeletal muscle will accelerate. It is reasonable to presume that if the adipogenic differentiation of MDSCs can be inhibited, the onset and progression of sarcopenia may be suppressed.

The application of low-magnitude high-frequency vibration (LMHFV), β-hydroxy-β-methylbutyrate (HMB) or both in sarcopenic mice could improve muscle mass and muscle function by reducing fat infiltration through the wnt/β-catenin signaling pathway [74, 75]. Lee et al. [76] demonstrated that fatty acid binding protein 3 (FABP3) upregulation could contribute to the age-associated loss of muscle mass and strength via modification of membrane lipid composition, which might promote endoplasmic reticulum stress and decrease protein synthesis, eventually leading to defective muscle mass and force. Hence, FABP3 may be a potential therapeutic target for sarcopenia intervention. In the field of TE to treat volumetric muscle loss defects, MDSCs, which adhere to scaffolds, can also play an important role, which has presented promising prospects for translating this method into the treatment of severe sarcopenia [77, 78]. Dong et al. [79] concluded that SW033291, a small-molecule inhibitor targeting 15-hydroxyprostaglandin dehydrogenase that subsequently elevates the production of prostaglandin E2 (PGE2), could promote the myogenic differentiation of MDSCs, which led to the repair of large skeletal muscle defects. Tamaki et al. [80, 81] successfully formed cardiomyocytes after myocardial infarction with the help of CD34( +)/CD45(−) MDSCs and suggested the possible future use of these cells for cardiac muscle repair in infarcted areas. Currently, the application of cocultured adipose-derived and muscle-derived stem cells suggests the best therapeutic value in stress-induced urinary incontinence compared with that of traditional single-cell methods by restoring the function and contractile force of the striated muscle of the sphincter [82]. Tamaki et al. [26] obtained MDSCs from the abdominal and leg muscles of 36 patients with ages ranging from 17 to 79 years. Unlike cells from mice, among these stem cells, two types of cells were shown to divide into highly myogenic (Sk-DN/29(+)) and multipotent stem cells (Sk-34). Then, the transplantation of these cells into the severely damaged muscles of athymic nude mice and rats was performed. Interestingly, mixed cultures of both cells resulted in reductions in tissue reconstitution capacities in vivo, whereas cotransplantation after separate expansion showed favorable results.

However, the regenerative capacity of skeletal muscle tissue is based on stem cells and their interplay with different muscle‐resident cell types within the niche [69]. Xu et al. [69], using a glycerol‐induced intramuscular fat infiltration mouse model as a research subject, concluded that a subpopulation of myeloid-derived cells may help increase intramuscular fat infiltration. It is worth noting that interstitial mesenchymal progenitors, where bone morphogenetic protein 3B (BMP3B) is specifically expressed, are responsible for fat infiltration in sarcopenic muscle [70]. The reduced expression of BMP3B is highly related to adipogenic differentiation, indicating that mesenchymal BMP3B expression maintains skeletal muscle integrity [70]. Depletion of mesenchymal progenitors can result in muscle weakness and atrophy [70]. An increase in macrophage number can enhance muscle regeneration function [37]. Thus, based on the studies to date, although we can affirm that stem cell-dependent therapies can counteract muscle aging, we may also need therapies targeting other types of cells together to obtain the best efficacy in this complex tissue with cellular heterogeneity.

### MDSCs and sarcopenia in relation to restoration of muscle mass

The loss of muscle mass is one of the notable features of sarcopenia. In general, reduced muscle mass or mild muscle damage can be repaired by SCs. However, there are several obstacles to the application of techniques simulating this process in clinical practice. Limited sources and the difficulty of isolating and purifying SCs are the primary problems. In contrast, the strengths of MDSCs are emerging. MDSCs can be expanded exponentially in vitro without losing proliferation or differentiation potential [32, 83]. In addition, MDSCs show good survivability under hypoxic and ischemic conditions [84]. SCs do not possess attractive properties that make MDSCs great candidates for stem cell-mediated therapy for sarcopenia. Tsao et al. [84] showed that MDSC implantation into the ischemic limb muscle of diabetic mice could reduce mortality, fat infiltration and apoptosis and increase stem cell number, myofiber central nuclei, and vascular and neural markers. MDSCs are a better option for the treatment of sarcopenia than SCs.

## Potential therapeutic strategies targeting stem cells to combat sarcopenia

Due to the great importance of skeletal muscle, someone with a disease manifesting as reduced muscle mass or function should be given high attention from physicians. Regrettably, there is not yet a consensus on sarcopenia management. It is widely accepted that preventing the occurrence and development of sarcopenia is the most important and simple way to combat this condition. According to the International Clinical Practice Guidelines for Sarcopenia (ICFSR) published in 2018 [85], resistance exercise training and dietary protein supplements are suggested strongly and conditionally, respectively. No specific drugs have been identified for the treatment of sarcopenia [2].

**Fig. 2:**
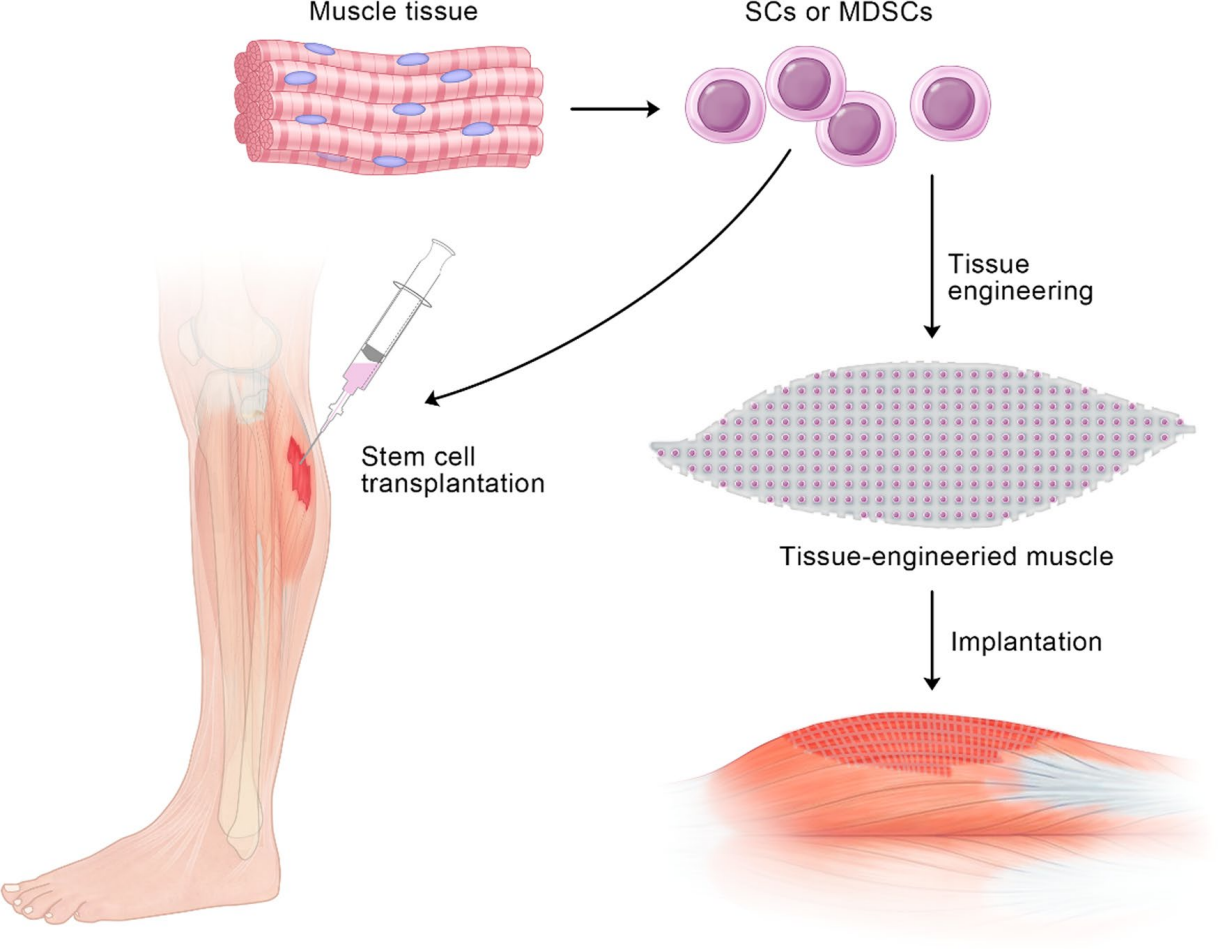
The graphical abstract of two therapy approaches based on stem cells for sarcopenia: TE and stem cell transplantation

It is the non‐dividing feature of mature myocytes that makes stem cells in muscle the only candidates for repair. To date, stem cell technology has been deeply explored and widely applied [86]. There have been few reports on its use in the treatment of sarcopenia, so we propose our own ideas for treatment strategies to combat sarcopenia, including tissue engineering (TE) and stem cell transplantation [87–89] (Fig. 2).

### TE

Stem cells in skeletal muscle could function as seed cells, an integral component of TE, for muscle regeneration [90]. Natural and synthetic materials, such as fibrin, alginate, polycaprolactone-based polymers and various other scaffold strategies, have been developed to generate skeletal muscle tissues in vitro [90, 91]. After stem cell isolation and purification to create an in vitro approximation of the in vivo stem cell niche and proliferation and differentiation in a simulated injury environment with proper cytokines, growth factors should be added to the blend of stem cells and scaffolds. Ascenzi et al. [92] suggested that insulin‐like growth factor‐1 (IGF‐1), which often appears in many anabolic pathways in skeletal muscle, could be a promising therapeutic agent for sarcopenia. Muscle mass supplementation provided by TE may treat weak diaphragm muscle caused by sarcopenia to prevent respiratory complications in aging individuals [93]. Many studies have proven that implantation of skeletal muscle tissue formed using TE in vitro into humans could enhance new myofiber formation within regional remaining muscle tissue. To date, the majority of in vitro TE strategies aim at creating functional skeletal muscle in the lab to offer new therapeutic possibilities for patients suffering from sarcopenia [94].

### Stem cell transplantation

It has been mentioned earlier in this article that the number of stem cells appears to be reduced and that function decreases in sarcopenic muscle. Therefore, we may particularly focus on restoring the age-related changes in stem cells in muscle by providing an extra supplement. To date, the efficacy of stem cell transplantation for treating muscle degenerative disease has been affirmed [95]. Collins et al. [96] showed that the transplantation of as few as seven satellite cells associated with one transplanted myofiber can generate over 100 new myofibers containing thousands of myonuclei while also undergoing self-renewal to sustain the endogenous stem cell pool. Even after transplantation of a single cell, it is capable of both producing copies of itself and generating more specialized progenitors, indicating the feasibility and efficiency of stem cell transplantation [97]. Regrettably, there have been few studies involving stem cell transplantation in clinically relevant muscle injury models.

### Exercise

With the rapid pace of modern work and life, a sedentary lifestyle has become common for numerous office workers, which increases the risk of sarcopenia later in life [98]. Hence, daily exercise and not sitting for long periods of time are very important. Regular physical exercise, regardless of whether it is aerobic or anaerobic or includes high or low resistance, has been considered an appropriate approach to combat many chronic or age-related diseases, such as sarcopenia [99, 100]. Age‐related muscle atrophy is due to a muscle fiber type‐specific decline in SC number and function [101]. Fortunately, exercise is able to restore SC quantity, and the acute SC response can be improved by prolonged exercise training in the elderly [101]. Encouragingly, Karlsen et al. revealed a robust capacity for adult regenerative myogenesis similar to young people after resistance training in healthy elderly human skeletal muscle [102]. Exercise can improve mitochondrial function [103]. Moreover, exercise may enhance autophagy in muscle, which is vital to muscle homeostasis and regeneration [66, 104, 105]. Joanisse et al. [106] showed that exercise significantly improved skeletal muscle regeneration in aging mice by increasing the activation and differentiation potential of stem cells [107]. Verdijk et al. [108] compared the degree of muscle capillarization in young and old people after 12 weeks of resistance exercise and found that this type of training could augment myofiber capillarization in older men. Shefer et al. [109] claimed that endurance exercise could protect SC numbers and that the reduction in myogenic capacity was induced by aging. Among the different modes, high-intensity interval exercise seems to be highly effective for SC pool expansion [110]. In response to exercise, Reddy et al. [111] demonstrated that human muscle secretes succinate through a pH-gated mechanism. Like many parts of the human body, SCs will preferentially stay in a quiescent state and risk turning into an irreversible nondividing state, which can severely threaten muscle regeneration after prolonged quiescence [112]. MDSCs could better participate in skeletal muscle repair because of the increased vascular endothelial growth factor (VEGF) secretion in a paracrine fashion caused by mechanical stimulation [113]. Thus, maintaining locomotion is highly beneficial to stem cells and skeletal muscle. Regardless of age, muscle retains the ability to positively respond to stimuli, such as exercise.

### Nutrition

Sarcopenia is becoming increasingly common in older people, and it is this group of people who show a high frequency of malnutrition in different contexts. Shen et al. [114] showed that nutrition combined with exercise has positive effects on obese elderly people with sarcopenia. Houston et al. [115] showed that lean mass in older adults varies with dietary protein intake. Kang et al. [116] demonstrated that whey protein supplementation brought significant improvements in muscle function in frail elderly individuals. Branched chain amino acids such as leucine may alleviate the decline in muscle mass, as reported by a systematic review and meta-analysis [117]. B-vitamins, vitamin D and many mineral elements have also been indicated to be effective [118–120]. A multicenter prospective longitudinal sarcopenia study, performed by Li et al. [121], showed that a 12-week intervention for elderly subjects consisting of intensive nutritional intervention and personalized designed resistance exercise can reduce the subclinical proinflammatory state and ameliorate sarcopenia. The conclusions of countless other articles also suggest that nutrition supplementation can be beneficial for patients with sarcopenia. Therefore, it is conceivable that managing nutrition well may help to prevent and treat sarcopenia.

## Concluding remarks and future directions

Sarcopenia is a disease characterized by progressive declines in muscle mass and function during aging among older people, and it is one of the major health problems in the elderly population, causing a huge burden on society and the health care system. Stem cell therapy for sarcopenia has been shown to promote muscle regeneration and repair in animal experiments. Exciting progress in preclinical and clinical studies has brought human stem cell therapy for newborn infants one step closer to clinical practice. However, the proper management of several issues, such as safety guarantees, uniform methods, standard diagnostic criteria, and improved understanding of detailed mechanisms, is required to permit the promotion of stem cell therapy. Furthermore, ethical requirements from countries worldwide inform this process, giving impetus to these novel therapeutic methods. In considering studies or clinical trials that have been finished thus far, we may hold the viewpoint that MDSC implantation is safe, as no negative effect has been reported from numerous rodent experiments, as well as preclinical and clinical human studies [19]. The number of current treatment methods for sarcopenia is limited, and the pathogenesis of this disease remains poorly understood. In future, more studies are needed to further unravel the association between stem cells in muscle and sarcopenia in terms of treatment and pathogenesis to improve the well-being of older patients. To date, most patients with sarcopenia have not been recognized and diagnosed. We believe that with clear diagnostic criteria and a reliable means of stem cell therapy in future, sarcopenia will be effectively controlled.

## Data Availability

Not applicable.
